# Preliminary Validation and Reliability Testing of the Montreal Instrument for Cat Arthritis Testing, for Use by Veterinarians, in a Colony of Laboratory Cats

**DOI:** 10.3390/ani5040410

**Published:** 2015-12-02

**Authors:** Mary P. Klinck, Pascale Rialland, Martin Guillot, Maxim Moreau, Diane Frank, Eric Troncy

**Affiliations:** 1 Québec Animal Pharmacology Research Group (GREPAQ), Department of Veterinary Biomedical Sciences, Faculty of Veterinary Medicine, Université de Montréal, 1500 des vétérinaires, Saint Hyacinthe, QC J2S 7C6, Canada; E-Mails: mary.klinck@umontreal.ca (M.P.K.); pascale.dh.rialland@gmail.com (P.R.); martin.guillot@yahoo.ca (M.G.); m.moreau@umontreal.ca (M.M.); 2 Department of Clinical Sciences, Faculty of Veterinary Medicine, Université de Montréal, 1500 des vétérinaires, Saint Hyacinthe, QC J2S 7C6, Canada; E-Mail: diane.frank@umontreal.ca

**Keywords:** metrology, degenerative joint disease, psychometric, pain measurement, behavior, osteoarthritis

## Abstract

**Simple Summary:**

Feline osteoarthritis (OA) is challenging to diagnose. A pain scale was developed for use by veterinarians, in association with their physical examination, and tested for reliability and validity. The scale items were: Interaction with the examiner, Exploration of the room, Body Posture, Gait, Body Condition, condition of Coat and Claws, and abnormal Findings or Cat Reaction upon joint Palpation. Expert review supported the scale content. Two studies using laboratory-housed cats found the most promising results for Gait and Body Posture, in terms of distinguishing between OA and non-OA cats, repeatability of results, and correlations with objectively measured kinetics (weight-bearing).

**Abstract:**

Subtle signs and conflicting physical and radiographic findings make feline osteoarthritis (OA) challenging to diagnose. A physical examination-based assessment was developed, consisting of eight items: Interaction, Exploration, Posture, Gait, Body Condition, Coat and Claws, (joint) Palpation–Findings, and Palpation–Cat Reaction. Content (experts) and face (veterinary students) validity were excellent. Construct validity, internal consistency, and intra- and inter-rater reliability were assessed via a pilot and main study, using laboratory-housed cats with and without OA. Gait distinguished OA status in the pilot (*p* = 0.05) study. In the main study, no scale item achieved statistically significant OA detection. Forelimb peak vertical ground reaction force (PVF) correlated inversely with Gait (Rho_s_ = −0.38 (*p* = 0.03) to −0.41 (*p* = 0.02)). Body Posture correlated with Gait, and inversely with forelimb PVF at two of three time points (Rho_s_ = −0.38 (*p* = 0.03) to −0.43 (*p* = 0.01)). Palpation (Findings, Cat Reaction) did not distinguish OA from non-OA cats. Palpation—Cat Reaction (Forelimbs) correlated inversely with forelimb PVF at two time points (Rho_s_ = −0.41 (*p* = 0.02) to −0.41 (*p* = 0.01)), but scores were highly variable, and poorly reliable. Gait and Posture require improved sensitivity, and Palpation should be interpreted cautiously, in diagnosing feline OA.

## 1. Introduction

Feline osteoarthritis (OA) has a high radiographic prevalence that increases with age [1], and is increasingly recognized as an important cause of pain and loss of physical function [2,3]. Improvements have been reported in mobility (e.g., jumping) and activity [4,5,6,7,8,9,10], lameness/stiffness [5,11], mood [6,9,11], and self-grooming [6] of cats with degenerative joint disease (DJD) including OA, in response to treatment with a non-steroidal anti-inflammatory drug, meloxicam [4,5,6,8,11] or robenacoxib [9], or a therapeutic diet [7]. One study found that OA cats receiving dietary supplementation with long-chain omega-3 fatty acids showed improved mobility *vs.* placebo [12]. However, the rate of diagnosis of this disease in the feline population appears to be low in relation to its radiographic prevalence [13], and physical examination findings (e.g., lameness, abnormalities upon palpation) do not necessarily correlate with radiographic signs [3,4,14]. Recent studies suggest that palpable abnormalities or pain are poorly sensitive for radiographic DJD in most joints (excepting the elbow, and lumbar and lumbosacral spine) [14], and the prevalence of radiographic signs in painful joints ranges from 33% [5] in one study to 85% in another [4]. Palpable abnormalities other than pain (e.g., decreased range of motion, joint thickening) are relatively uncommon in joints other than the elbow [4,5] and the hock [4]. One study found that palpation findings did not agree with historical signs of pain but did agree moderately with thermographic findings [15]. It appears that veterinarians rely heavily on owner-reported abnormalities to diagnose OA; diagnosis was rare in the absence of anamnestic signs in one study, and even cases with historical signs often lacked abnormalities upon palpation [16].

Individual subject differences in pain experience and expression, and differences in examiner interpretive capabilities, are challenges in pain assessment. Objective measures such as peak vertical ground reaction force (PVF) [17,18,19,20], von Frey punctate tactile withdrawal threshold (VF) [17], telemetered locomotor activity monitoring (AM) [5,17,19], thermographic imaging [15], functional bio-imaging [21], kinematics [22] and response to mechanical temporal summation [23] showed promise for detecting OA pain. However, these measures may not be feasible in clinical practice. In one study, AM did not distinguish OA from non-OA cats (due to high inter-individual variability), despite detection of treatment effects [17]. Pain scales offer a relative objectivity and facilitate comparison within and between individuals [24]. Recent research has sought to develop and validate owner pain scales for feline OA; examples include a client-specific outcome measure questionnaire [5] and two standardized multi-item numerical rating scales (NRSs) [6,25,26,27]. Scale validation is needed to confirm effective and consistent measurement of the condition of interest, in the target context. This process comprises multiple aspects addressed in separate experiments [28]. “Content” validation assesses scale completeness and representativeness, generally via expert review; “face” validation comprises a similar assessment by a naïve population and also relates to acceptability of the scale [28,29]. “Criterion” validation uses a “gold standard” to evaluate scale performance, either at the same (“concurrent”) or a later (“predictive”) time [28]. “Construct” validation evaluates how well the scale measures the condition of interest (e.g., pain) when direct quantification using a gold standard is impossible (e.g., for subjective experiences like pain or anxiety). It involves hypothesis testing (e.g., between-groups comparison), and determination of convergence with related (e.g., motor function), and divergence from distinct (e.g., fear) constructs [28,29]. “Reliability” refers to degree of freedom from measurement error, which includes inter- (scoring consistency between evaluators) and intra-rater (scoring stability for one evaluator over time) reliability, and internal consistency (interrelatedness of scale items) [28,29]. 

The objectives of this study were to develop a scale for use by veterinarians in conjunction with the physical examination, to perform content and face validation, and to assess both its ability to detect OA pain, and its reliability. We hypothesized that a standardized assessment combining examination room behavior, distance evaluation of posture and gait, and hands-on examination of body condition, and joint palpation and manipulation, could be used to detect clinical OA in cats.

## 2. Experimental Section

### 2.1. Materials and Methods

All study protocols were approved by the Institutional Animal Care and Use Committee (# Rech-1482). The guidelines of the Canadian Council on Animal Care were followed regarding cat care and handling.

### 2.2. Part I: Scale Development, and Content and Face Validity

Preliminary content for the Montreal Instrument for Cat Arthritis Testing–Veterinarian (MI-CAT(V)) was developed based on a review of the literature and the authors’ collective experience. The resulting eight items were each ranked on an NRS (ranging from zero to between two and six, where zero = normal). The proposed assessment was in two parts. The first was a distance observation including (1) Interactive Behavior with respect to the examiner, (2) Exploratory Behavior in the examination room, (3) Posture, and (4) Gait. The second was a hands-on evaluation of (5) Body Condition Score, (6) Coat and Claws (condition), (7) Joint Palpation (including manipulation)–Findings (e.g., altered range of motion, crepitus, joint thickening, muscle atrophy), and (8) Joint Palpation (including manipulation)—Cat Reaction (e.g., vocalization, withdrawal, tension, biting, scratching).

Content validity was tested via an internal (*n* = 3; MPK, DF, ET) and an external (*n* = 4; international experts in feline pain) evaluation. Each reviewer ranked scale items on their clarity, importance, and the appropriateness of response options (possible ranks of one to three; one = poor, two = fair, three = good), and commented on specific items, and general scale construction and content. Subsequent scale modifications included: (1) minor reordering of items evaluated via distance observation (Exploratory Behavior followed by Gait, Posture, then Interactive Behavior), (2) wording changes to improve clarity, and (3) expansion of the Posture, Coat and Claws, Palpation–Findings, and Palpation–Cat Reaction items. (See Figure S1: MI-CAT(V)-v1).

Face validity consisted of a similar assessment by third-year veterinary students (*n* = 80). Respondent gender was noted, as well as whether they had ever: (1) owned a cat, (2) considered that cats could develop OA, and (3) assessed animal pain (acute or chronic). No subsequent scale modifications were made.

### 2.3. Part II: Reliability Assessment and Construct Validity

Evaluation of scale inter- and intra-rater reliability, internal consistency, and construct validity (distinction between OA and non-OA cats, concordance with functional and neurophysiological tests) was conducted in two phases, a pilot and a main study.

All cats were group-housed in dedicated, environmentally controlled rooms beginning four weeks prior to each study. A standard certified commercial diet (Hill’s Prescription Diet^®^ w/d^®^ Feline, Hill’s Pet Nutrition, Inc^®^, Mississauga, ON, Canada) was fed once daily, according to manufacturer recommendations, and water was supplied free choice. Cats could move freely about the rooms at all times, and enrichment was provided in the form of toys, windows, and climbing, perching and hiding areas.

Screening for OA consisted of complete physical examination, and digital radiography (DR; mediolateral and caudocranial views of stifle, coxofemoral, lumbosacral, sacroiliac, carpal and tarsal joints, and mediolateral views of shoulders and elbows). Cats were sedated for DR with intramuscular medetomidine (0.02 mg/kg; Domitor 1 mg/mL, Zoetis Canada, Kirkland, QC, Canada) and morphine (0.1–0.2 mg/kg; Morphine Sulfate Injection 10 mg/mL, Sandoz, Boucherville, QC, Canada). Inclusion criteria specific to OA cats were: (1) OA-related radiographic changes (osteophytosis, subchondral bone sclerosis, and/or joint surface remodeling) [17] with (2) orthopedic examination findings consistent with OA in the radiographically affected joint(s). Inclusion criteria for non-OA cats were: (1) the absence of OA-related radiographic changes in all joints assessed, and (2) a normal orthopedic examination. Cats could neither have received analgesic, anti-inflammatory or potential structuro-modulator (e.g., glucosamine) medications during the three months prior to the study, nor have any clinically significant abnormalities other than OA on physical examination, complete blood count, blood chemistry (including T4), and urinalysis.

All MI-CAT(V) assessments were performed in a 1.5 × 3.9 m room with a 2-level (38 cm and 90 cm) examination table; cats were encouraged to move about and to jump up and down by calling, tossing treats or toys, petting, or brushing. Raters were blinded to cat OA status.

The pilot study included seven neutered, adult, domestic cats with both structural changes and orthopedic examination findings consistent with coxofemoral OA, and four with neither radiographic nor orthopedic examination findings consistent with OA in any joint. MI-CAT(V) evaluations were performed concurrently by two examiners (MK and PR) on Days zero, seven, 32 and 35; only MK (the reference observer) performed joint palpation and manipulation. Scale modifications based on the results included reorganization and expansion of the response options for Exploratory Behavior and Gait. Additionally, Body Condition, Coat and Claws, and Palpation–Findings were removed, and the response options for Palpation–Cat Reaction were simplified. (See Figure S2: MI-CAT(V)-v2).

For the main study, 120 neutered, adult domestic cats underwent OA screening. Thirty-eight met inclusion criteria and were retained: 32 OA (19 females and 13 males; mean (SD) of 8 (2.4) years), and six non-OA cats (three females and three males; 2.8 (1.4) years). Joints affected in the OA cats were, in order of decreasing prevalence: the hip (*n* = 21, 65.6%), shoulder (*n* = 12, 37.5%), tarsus (*n* = 12, 37.5%), stifle (*n* = 11, 34.4%) elbow (*n* = 9, 28.1%), and carpus (*n* = 4, 12.5%). Most cats had one affected joint in the forelimb, this number ranged from 0 to 4. In the hind limb, the median number of radiographically OA-affected joints was 2 with a range of 0 to 4. The two raters assessed a subset of the screened cats (*n* = 27) prior to the study start (day-34), each using the entirety of the revised scale, to evaluate its inter-rater reliability.

Prior to the study, cats were trained with food treats to traverse a pressure-sensing walkway, and were habituated to a von Frey test cage. One veterinarian (MPK) performed three, weekly scale assessments (days zero, seven and 14). Intra-rater reliabilities were calculated based on comparisons between pairs of days, and internal consistency and ability to detect OA were assessed for each day. Scale convergent (construct) validity was evaluated using the following tests: (1) PVF via a floor mat-based plantar force measurement system (Walkway® with Matscan® WE5 sensors, Tekscan, Boston, MA, USA), and (2) secondary punctate allodynia/hyperalgesia response via electronic VF withdrawal threshold measurements (Rigid Tip, 0.7 mm^2^, 28 G; IITC Life Science, Woodland Hills, CA, USA), as previously described [19]. 

### 2.4. Statistical Analyses

Intra- and inter-rater reliabilities were evaluated using Spearman’s rank correlation (systematic biases) and a weighted Kappa for multiple categories (agreement) [30,31]. Interpretation of Kappa was based on that previously described, as follows: ≤0.00 = poor, 0.01–0.20 = slight, 0.21–0.40 = fair, 0.41–0.60 = moderate, 0.61–0.80 = substantial, 0.81–1.00 = almost perfect [32]. Spearman’s rank correlations between individual scale item scores were used to evaluate internal consistency; they were also used to compare PVF and VF outcomes with scale scores. Interpretation of these correlations was as follows: 0–0.35 = weak, 0.36–0.70 = moderate, 0.71–1.00 = strong. The scale’s ability to distinguish between OA and non-OA cats was tested using exact Wilcoxon-Mann-Whitney tests. All analyses were two-tailed with an α-level of 0.05, and analyses were performed using statistical software (SAS^®^ system, version 9.2, SAS Institute Inc., Cary, NC, USA; JMP^®^, Version 9, SAS Institute Inc., 2010; Stata^®^ Statistical Software, release 12, StataCorp LP. College Station, TX, USA, 2011).

## 3. Results 

### 3.1. Part I: Scale Content and Face Validity

For the expert review, the median global item scores (*i.e.*, sum of clarity, importance, and appropriateness of response options scores) were either eight or nine out of nine for all items. A variety of comments on content and presentation were made, and these were incorporated into the MI-CAT(V)-v1 as described above.

Out of 80 students, 77 completed the review (96.3%). Many had owned cats (*n* = 62, 80.5%) or reported previously having evaluated pain in animals (*n* = 64, 83%); whether acute (*n* = 53, 68.8%) or chronic (*n* = 33, 42.9%). Forty-six (59.7%) had previously considered that cats could develop OA. All median global item scores were nine out of nine, except for Interactive Behavior (eight out of nine). Comments on content and presentation were varied, the most common being that individual temperament differences might affect the Exploratory Behavior and Interactive Behavior items, and that Gait might not be evaluable in a clinic setting.

**Figure 1:**
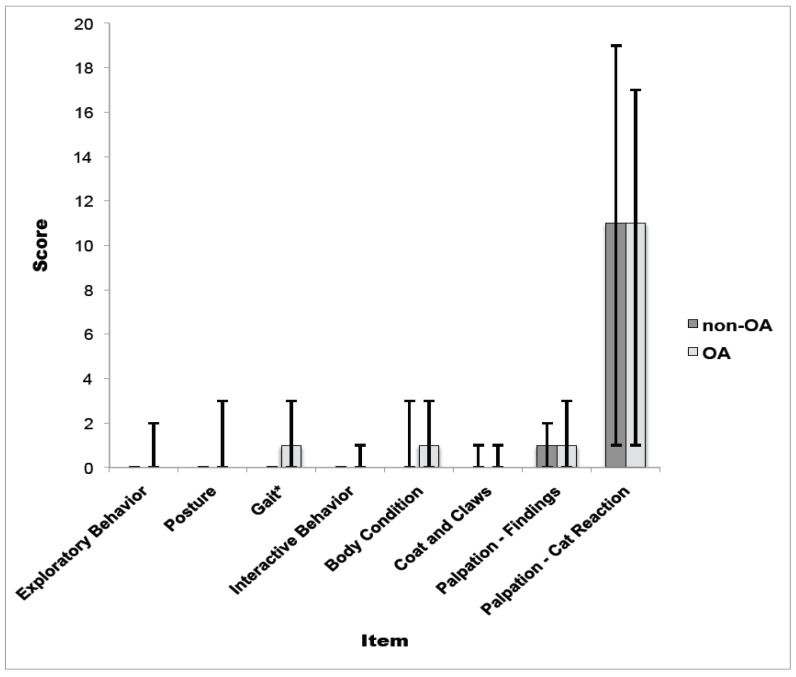
MI-CAT(V)-v1 scores by scale item for OA (*n* = 7) *vs.* non-OA (*n* = 4) cats (Pilot Study Day 0). Medians are presented (error bars denote minimum and maximum). * *p* = 0.05.

### 3.2. Part II: Reliability Assessment and Construct Validity

For the pilot study (MI-CAT(V)-v1), all items on the scale, except Palpation—Cat Reaction, tended to be assessed at the low end of possible scores: scores of zero were frequent. Only Gait distinguished between cats with and without OA (*p* = 0.05). (See Figure 1) Intra-rater reliability was fair to excellent for all items but Gait, Palpation—Findings, and Palpation—Cat Reaction. (See Table 1) Inter-rater reliability improved from the first (Day zero) to the second (Day 35) evaluation, when it was fair to excellent for all items. (See Table 2) There were significant correlations between: (1) Exploratory Behavior and Interactive Behavior (Spearman’s Rho (Rho_S_) = 0.74 (*p* = 0.01)), (2) Exploratory Behavior and Body Posture (Rho_S_ = 0.79 (*p* < 0.001)), (3) Body Posture and Gait (Rho_S_ = 0.83 (*p* < 0.001)), (4) Body Condition and Coat and Claw Condition (Rho_S_ = 0.69 (*p* = 0.02)), and (5) Palpation—Findings and Palpation—Cat Reaction (Rho_S_ = 0.68 (*p* = 0.03)). 

**Table 1:** Intra-rater reliability for the Montreal instrument for cat arthritis testing-veterinarian-version 1 (MI-CAT(V)-v1) tested first at baseline over one week, and again over three days approximately one month later (*n* = 11 cats, 7 osteoarthritis (OA), 4 non-OA).

Scale Item	Days 0 and 7		Days 32 and 35	
	Kappa	Rho_s_	Kappa	Rho_s_
Exploratory Behavior	0.83	0.82 (*p* < 0.0001)	0.33	0.64 (*p* = 0.03)
Body Posture	0.64	0.60 (*p* = 0.05)	0.64	0.71 (*p* = 0.01)
Gait/Locomotion	−0.11	NS	−0.11	NE
Interactive Behavior	1.00	1.00 (*p* < 0.0001)	1.00	1.00 (*p* < 0.0001)
Body Condition	0.37	0.65 (*p* = 0.03)	0.37	1.00 (*p* < 0.0001)
Coat and Claws	0.80	0.82 (*p* < 0.0001)	0.80	NS
Palpation—Findings	0.18	NS	0.18	0.62 (*p* = 0.04)
Palpation—Cat Reaction	0.32	0.64 (*p* = 0.04)	0.32	0.64 (*p* = 0.04)

Legend: Rho_s_ = Spearman’s rho; NS = not significant; NE = not evaluable.

**Table 2:** Inter-rater reliability (MI-CAT(V)-v1) for two observers tested on two occasions (*n* = 11 cats, 7 OA and 4 non-OA).

Scale Item	Day 0		Day 35	
	Κappa	Rho_s_	Kappa	Rho_s_
Exploratory Behavior	0.37	NS	0.84	0.85 (*p* < 0.01)
Body Posture	−0.41	NS	0.32	0.67 (*p* = 0.02)
Gait/Locomotion	0.10	NS	0.33	0.81 (*p* < 0.01)
Interactive Behavior	0.33	0.64 (*p* = 0.03)	0.62	0.67 (*p* = 0.02)
Body Condition	0.38	NS	0.52	0.59 (*p* = 0.05)
Coat and Claws	NE	NE	1.00	1.00 (*p* < 0.01)
Palpation—Cat Reaction	0.55	0.94 (*p* < 0.01)	0.68	0.86 (*p* < 0.01)

Legend: Rho_s_ = Spearman’s rho; NS = not significant; NE = not evaluable.

Four cats were withdrawn during the main study, three OA (one each due to vestibular syndrome, severe recurring diarrhea, and fear and aggression associated with handling), and one non-OA cat (due to unreliable responses to functional/neurophysiologic testing). Osteoarthritis was especially prevalent in the hindlimbs: 27 of 29 OA cats had at least one affected hindlimb joint. The coxofemoral (18/27 = 66.7%), tarsal (8/27 = 29.6%), and stifle (6/27 = 22.2%) joints were affected. The MI-CAT(V) evaluation took approximately 10–15 minutes per cat. Intra-rater reliability was best for Exploratory Behavior and Interactive Behavior, and acceptable for Body Posture with lower reliability for hind limb Posture. It was somewhat less good for Gait, and still less so for Reaction to Palpation. (See Table 3) Inter-rater reliability for the MI-CAT(V)-v2 was best for Interactive Behavior, Exploratory Behavior, and Body Posture (specifically, Posture of the hind limbs). The Gait assessment was poorly reproducible. Reliability results for Reaction to Palpation were heterogeneous, but acceptable for the hind limbs. (See Table 4) There were significant correlations at all time points between: (1) Exploratory Behavior and Interactive Behavior (Rho_S_ = 0.42 (*p* = 0.01) to 0.58 (*p* = 0.0003)), (2) Gait and Body Posture (Rho_S_ = 0.65 (*p* < 0.0001) to 0.73 (*p* < 0.0001)), and (3) Palpation–Cat Reaction and each of Exploratory Behavior (Rho_S_ = 0.54 (*p* = 0.0009) to 0.58 (*p* = 0.0004), Gait (Rho_S_ = 0.77 (*p* < 0.0001) to 0.83 (*p* < 0.0001)), and Body Posture (Rho_S_ = 0.83 (*p* < 0.0001) to 0.88 (*p* < 0.0001)).

**Table 3:** Intra-rater reliability (MI-CAT(V)-v2) tested on three occasions, at one week intervals, (*n* = 34 cats, 29 OA and 5 non-OA).

Scale Item	Day 0–Day 7		Day 7–Day 14		Day 0–Day 14	
	Kappa	Rho_s_	Kappa	Rho_s_	Kappa	Rho_s_
Exploratory Behavior	0.41	0.60 (*p* = 0.0002)	0.43	0.60 (*p* = 0.0002)	0.60	0.69 (*p* < 0.0001)
Body Posture						
Axial	0.58	0.72 (*p* < 0.0001)	0.69	0.86 (*p* < 0.0001)	0.84	0.85 (*p* < 0.0001)
Forelimbs	0.36	0.48 (*p* = 0.0041)	0.44	0.54 (*p* = 0.001)	0.40	0.40 (*p* = 0.0176)
Hind limbs	0.51	0.62 (*p* < 0.0001)	0.18	NS	0.57	0.74 (*p* < 0.0001)
*Posture Total*	0.40	0.69 (*p* < 0.0001)	0.40	0.57 (*p* = 0.0004)	0.54	0.78 (*p* < 0.0001)
Gait	0.33	0.68 (*p* < 0.0001)	0.38	0.45 (*p* = 0.0072)	0.28	0.54 (*p* = 0.0011)
Interactive Behavior	0.57	0.66 (*p* < 0.0001)	0.78	0.86 (*p* < 0.0001)	0.48	0.55 (*p* = 0.0008)
Reaction to Palpation						
Axial	NE	0.62 (*p* < 0.0001)	NE	0.62 (*p* < 0.0001)	NE	0.65 (*p* < 0.0001)
Forelimbs	0.26	0.72 (*p* < 0.0001)	0.36	0.52 (*p* = 0.0018)	0.38	0.56 (*p* = 0.0006)
Hind limbs	0.25	0.68 (*p* < 0.0001)	0.23	0.48 (*p* = 0.004)	0.44	0.75 (*p* < 0.0001)
*Palpation Total*	−0.02	0.72 (*p* < 0.0001)	0.00	0.63 (*p* < 0.0001)	NE	0.72 (*p* < 0.0001)

Legend: Agreement is assessed between each pair of evaluation days. Rho_s_ is Spearman’s rho; NS = not significant; NE = not evaluable.

**Table 4:** Inter-rater reliability (MI-CAT(V)-v2) for two observers tested on one occasion (*n* = 27 cats, 22 OA and 5 non-OA).

Scale Item	Kappa	Rho_s_
Exploratory Behavior	0.45	0.55 (*p* < 0.0001)
Body Posture		
Axial	0.31	NS
Forelimbs	−0.02	NS
Hind limbs	0.44	0.42 (*p* = 0.0014)
*Posture Total*	0.41	0.42 (*p* = 0.0012)
Gait	0.22	NS
Interactive Behavior	0.55	0.60 (*p* < 0.0001)
Reaction to Palpation		
Axial	0.24	0.29 (*p* = 0.0306)
Forelimbs	0.03	NS
Hind limbs	0.35	0.38 (*p* = 0.0035)
*Palpation Total*	0.21	NS

Legend: Rho_s_ is Spearman’s rho; NS = not significant; NE = not evaluable.

No statistically significant differences were found between OA and non-OA cats for any scale items. Medians and ranges for individual items suggested non-significant trends for two, Gait and Body Posture, to yield higher (*i.e.*, worse) scores for cats with OA. Reaction to Palpation, particularly of the hind limbs, tended to yield higher scores for non-OA cats. (See Table 5) Exploratory Behavior and Interactive Behavior did not appear to be sensitive to OA status.

**Table 5:** MI-CAT(V)-v2 scores by scale category for OA (n = 29) *vs.* non-OA (n = 5) cats, by evaluation time.

Scale Item	Day 0		Day 7		Day 14	
	OA	Non-OA	OA	Non-OA	OA	Non-OA
Exploratory Behavior	1 (1–6)	1 (0–5)	1 (0–6)	1 (0–5)	1 (0–5)	1 (0–5)
Body Posture						
Axial	0 (0–1)	0 (0)	0 (0–2)	0 (0–1)	0 (0–1)	0 (0)
Forelimbs	0 (0–1)	0 (0)	0 (0–2)	0 (0)	0 (0–1)	0 (0)
Hind limbs	1 (0–2)	0 (0–1)	0 (0–2)	0 (0)	0 (0–2)	0 (0–2)
*Posture Total*	1 (0–3)	0 (0–1)	1 (0–3)	0 (0–1)	1 (0–2)	0 (0–2)
Gait	3 (0–5)	1 (0–3)	3 (0–5)	0 (0–3)	3 (0–5)	2 (0–4)
Interactive Behavior	0 (0–2)	0 (0–1)	0 (0–1)	0 (0–2)	0 (0–1)	0 (0–1)
Reaction to Palpation						
Axial	1 (0–5)	1 (0–3)	1 (0–5)	1 (0–2)	1.5 (0–4)	2 (0–3)
Forelimbs	2 (0–4)	3 (1–3)	2 (0–4)	3 (1–4)	2 (0–4)	3 (2–4)
Hind limbs	5 (0–8)	6 (3–7)	5 (1–9)	5 (2–6)	5 (2–7)	5 (4–6)
*Palpation Total*	5 (0–8)	6 (3–7)	5 (1–9)	5 (2–6)	5 (2–7)	5 (4–6)
Scale Total	9 (2–17)	11 (4–13)	8 (3–18)	10 (2–14)	9 (5–13)	10 (3–16)

Legend: Values are presented as median (range).

Von Frey and PVF distinguished between OA and non-OA cats, as reported elsewhere [19]. Based on VF data distribution, an allodynia threshold was set at 40 g for the front paws and 50 g for the hind paws. These thresholds were determined based on the first quartile values of the OA cats, and no non-OA cat had such low values. Twenty-five percent of OA cats were allodynic *vs.* none of the non-OA cats. No associations were found between scale and VF assessments. There were moderate negative correlations between forelimb PVF and Gait at all time points (Rho_S_ = −0.38 (*p* = 0.03) to −0.41 (*p* = 0.02)). On days seven and 14, a moderate negative correlation was also found between Body Posture (Forelimbs) and forelimb PVF (Rho_S_ = −0.38 (*p* = 0.03) to −0.43 (*p* = 0.01)), and between Reaction to Palpation (Forelimbs) and forelimb PVF (Rho_S_ = −0.41 (*p* = 0.02) to −0.41 (*p* = 0.01)).

## 4. Discussion

The use of a combination of historical, orthopedic examination, performance test and radiographic findings has been recommended to reduce the uncertainty associated with feline OA diagnosis [2]. However, there is clearly a mismatch between physical examination findings and radiographic signs of OA [4,5,14], as well as between historical and orthopedic examination findings [12,16], and it is not evident just what performance tests may be most effective for detecting OA pain in clinical practice. Although owner pain scales for feline OA have recently been described [5,6,25,26,27], interpretation of physical examination findings in at-risk patients requires better understanding. This study attempted to determine the most reliable and valid examination procedures (including performance tests) for feline OA detection.

The MI-CAT(V) performed well in the initial naïve and expert reviews, supporting its content. Based on evaluation of reviewer comments, the existing literature, and the preliminary reliability and construct validity assessed via the pilot study, the items appearing least promising, Body Condition, Coat and Claws, and Palpation–Findings, were removed. The only item capable of detecting OA was Gait; none of the former distinguished OA from non-OA cats, nor were they correlated with Gait. Body Condition was variable in all cats and less reliable than anticipated; previous reports do not establish a clear relationship with musculoskeletal disease [33,34]. Other diseases and owner intervention may have a substantial effect on both Body Condition and Coat and Claws (e.g., diet changes, brushing and claw-trimming). These physical aspects may therefore be more suitably assessed using an owner scale, and hence were not retained for further evaluation in the main study. Palpation—Findings other than pain were rare in the pilot study, consistent with other studies [4,33]; this item was therefore also eliminated prior to the main study. Of the retained items, Gait, Body Posture, Exploratory Behavior and Interactive Behavior demonstrated inter-item correlations in the pilot study, and Gait detected OA. The association between Exploratory Behavior and Interactive Behavior suggests convergence of these items, possibly evaluating the effects of chronic painful disease on cat temperament, whereas convergence of Body Posture and Gait would appear to be associated with biomechanical alterations and pain. Interestingly, Gait’s intra-rater reliability was poor in the pilot study, but its inter-rater reliability was good at the second set of scale assessments. Given that inter-rater reliability of all items improved as the second veterinarian became more familiar with the scale (training effect), this may suggest that Gait had poor stability (*i.e.*, that it varied between assessment days). Gait was therefore revised in an attempt to improve its stability and sensitivity. Body Posture, Exploratory Behavior, and Interactive Behavior showed non-significant trends toward distinguishing OA from non-OA cats in the pilot study and were retained for further testing in the main study. Palpation–Cat Reaction performed poorly in the pilot study, with respect to sensitivity to OA; however, given the clinical reliance on joint palpation as a diagnostic tool, it was revised and retained for further evaluation.

It was disappointing that no scale items distinguished OA from non-OA cats in the follow-up evaluation (MI-CAT(V)-v2), but, once again, Gait appeared the most promising for detecting OA signs. It may be that the presence of multiple affected joints/limbs makes postural and gait abnormalities more difficult to detect in feline OA. This apparent lack of sensitivity might be corrected in the future via alterations to the weights of these items. It is of note that these criteria, particularly Gait, are related to mobility/activity [4,5,6,7,8,9,10] and lameness/stiffness [5,11], which have previously been described as sensitive to OA and responsive to anti-inflammatory treatment. However, reliability was generally good. Although both VF and PVF distinguished between OA and non-OA cats [19], we found correlations only between PVF and scale items, specifically, lower forelimb PVF was associated with increased (*i.e.*, abnormal) Gait, Body Posture (Forelimbs) and Palpation–Cat Reaction (Forelimbs) scores. The fact that most of the OA cats were affected in the hind limbs suggests that behavioral expression might be accentuated in the compensating (forelimb in the study) limbs. It appears that scale items did not detect hyperalgesia/allodynia, but may have detected functional changes (PVF), associated with OA. It should be noted that the presence of allodynia in 25% of OA cats, stable over time and unresponsive to meloxicam treatment [19], holds promise for neurophysiological pain assessment in the future. In the present study, none of the MI-CAT(V) scale items were constructed to detect this hypersensitivity. The item most expected to have done so would have been Reaction to Palpation, and this item was only associated with Exploratory Behavior, Gait and Body Posture. The relationship of the latter two suggests that they reflect some degree of biomechanical pain. Exploratory Behavior and Interactive Behavior, on the other hand, might be influenced more by the global temperament of the cat, reflecting neurophysiological changes and its individual experience of pain. It was particularly interesting that joint palpation and manipulation largely failed to distinguish OA from non-OA cats. This was despite cat selection based in part on an apparently painful response to palpation, and did not change whether the assessment was subjective (*i.e.*, was there a painful response) or objective (*i.e.*, did the cat flinch, withdraw, vocalize, *etc.*). It is of note that, despite correlations with other scale items (Gait, Body Posture, and Interactive Behavior) and the correlation with forelimb PVF, Palpation–Cat Reaction actually had tendency for higher scores in non-OA than in OA cats, in contrast to a previous study’s findings [14]. This striking finding may explain the great difficulty in validating this type of response measure, and is potentially influenced by stimulus heterogeneity, subject temperament, and stress. On the basis of these results, the use of cat reaction to palpation cannot be recommended for clinical OA diagnosis. The authors are aware of no other studies of inter and intra-rater reliability of joint palpation findings in cats, and it may be that this method suffers due to poor reliability, or that it is simply not valid due to the effects of cat factors other than pain (e.g., temperament). In any case, our findings call into question the emphasis to be placed on the findings of joint palpation and manipulation for evaluating feline OA. New developments addressing tactile hypersensitivity in OA cats are promising, such as the response to mechanical temporal summation [23]. They now need to be translated into clinical assessment.

It should be noted that this research was conducted in a laboratory colony of cats, and that this may influence applicability of the results to the clinical context. These cats became familiar with the evaluators and the environment over the course of the study, and lacked some of the stressors present in clinical patients (e.g., car travel, presence of unfamiliar animals). This may have influenced their behavior and facilitated assessment in the laboratory context. Cats may show inhibited or aggressive behavior in a clinical setting, due to fear, and, in the clinic, it is possible that this may be more of a problem in a population of client-owned cats. Future evaluation of the MI-CAT(V) will need to be performed to determine its feasibility in cats visiting a veterinary clinic. That being said, care was taken to make the environment similar to that of an examination room, the cats were not initially familiar with the room, scale procedure, or raters, and the evaluation itself was not so lengthy as to preempt its use in practice.

A frequent problem in the validation of pain scales is the lack of a true “gold standard” for measuring pain [28]. We compared cats with both radiographic and physical examination findings consistent with OA, to cats with neither. Misclassification would have been possible given the difficulties inherent in the physical examination of feline OA, and the incomplete concordance between radiographic and clinical OA. However, this method was expected to yield a high index of suspicion for OA *vs.* non-OA status. The addition of concurrent functional (PVF) and neurophysiological (VF) evaluations related to OA [17,18,19] yielded a multifaceted approach to construct validation.

## 5. Conclusions

Based on the preliminary evaluation of the MI-CAT(V), a pain scale for use by veterinarians, it is concluded that the role of limb palpation in the diagnosis of feline clinical OA remains unclear; caution is urged in its interpretation. While body condition, and condition of coat and claws may be altered in OA, these should be assessed in light of the owner’s interventions. The items assessing gait/locomotion, and possibly body posture, showed the most promise, but would benefit from further refinement to increase sensitivity and reliability in order to determine whether they can actually differentiate between cats with and without OA. They would then require evaluation for responsiveness to treatment, feasibility and usefulness in guiding treatment decisions, in client-owned animals. The importance of owner pain scales for use in feline OA is reaffirmed, given the uncertainties that remain regarding the interpretation of physical examination findings.

## Supplementary Files

Supplementary File 1
