# Supplementary material for: Preliminary Validation and Reliability Testing of the Montreal Instrument for Cat Arthritis Testing, for Use by Veterinarians, in a Colony of Laboratory Cats
Source: Animals (Basel). 2015 Dec 2;5(4):1252–67. doi: 10.3390/ani5040410 (PMC4693214; doi:10.3390/ani5040410)
Supplement: Supplementary File 1 [file animals-05-00410-s001.pdf]

Preliminary Validation and Reliability Testing of the Montreal Instrument for Cat Arthritis Testing, for Use by Veterinarians, in a Colony of Laboratory Cats

MI-CAT(V) – Montreal Instrument for Cat Arthritis Testing (Veterinary)

#	Category	Assessment Criteria	Grade
Assign a value for each of categories 1-4 prior to hands-on examination. The cat should be allowed to walk on exam room floor, be placed on a low bench or chair to observe willingness/ability to jump down, and encouraged to jump up by placing the empty carrier on a bench/chair in front of the cat.			
1	Exploratory Behavior	Walks, runs or jumps freely	0
		Walks slowly/cautiously, or with abnormal or lowered body posture	1
		No ambulation/exploratory behavior	2
2	a. Body Posture – **head, torso, tail**	Ambulates/stands/sits/lies with even weight distribution from front to rear, back level, head up, tail above horizontal	0
		Head low/tail lowered (not tucked)	1
		Overt abnormalities: weight shifted forward or backward, hunched back, limp tail<br>- 1 finding	2
		- ≥2 findings	3
	b. Body Posture – **front limbs**	Ambulates/stands/sits/lies with limbs in normal state of flexion/extension, even weight distribution from right to left	0
		Overt abnormalities: limb hyperflexion, limb hyperextension, unequal weight distribution from right to left, or other asymmetry<br>- 1 finding	1
		- ≥2 findings	2
	c. Body Posture – **rear limbs**	Ambulates/stands/sits/lies with limbs in normal state of flexion/extension, even weight distribution from right to left	0
		Overt abnormalities: plantigrade stance, limb hyperflexion or hyperextension, unequal weight distribution from right to left, or other asymmetry<br>- 1 finding	1
		- ≥2 findings	2
3	Gait/Locomotion	Normal gait, jumps up/down willingly and smoothly	0
		Normal gait, reluctant or unwilling to jump	1
		Generally normal gait, occasionally awkward (e.g., misses a jump or missteps)	2
		Mildly to moderately abnormal gait (e.g., stiff or weak, or with abnormal limb placement or carriage)	3
		Obviously limping on 1 or more limbs	4

Figure S1. *Cont.*

4	Interaction with Examiner	Friendly: approaches/rubs/wants to be petted	0
		Immobile: neither avoids nor solicits contact	1
		Withdraws/avoids touch	2
		Hisses/growls/swats/bites or threatens	3
5	Body Condition Score	Normal: 3/5	0
		Thin: 1/2-2/5	1
		Overweight: 4/5	1
		Obese: 5/5	2
6	a. Coat Condition	Clean, shiny, no mats	0
		Unkempt, flaky, not shiny; no overt dirt/mats	1
		Dirty/matted (localized)	2
		Dirty/matted (generalized)	3
	b. Claw Condition<br>Note overgrown claws (not abnormal claws in polydactyls).<br>Note "0" if all feet declawed.	Claws sharp (unless trimmed), normal length/thickness	0
		Occasional overgrown (thick or excessively long) claw	1
		Most claws on one or more paws overgrown	2

For categories 7 and 8, perform palpation and manipulation (including full flexion/extension, as well as assessment of side-to-side and cranial-caudal motion) of the neck, back, tail, and each appendicular joint, with the patient standing first, then in lateral recumbency. Do not repeat manipulation if patient attempts to bite.

7 Palpation and Manipulation – **Findings** (check ALL that apply)

Joint	Muscle atrophy	Joint thickening	Crepitus	Reduced range of motion	Total for each joint (out of 4)
Cervical spine					/4
Thoracolumbar spine					/4
Sacrococcygeal spine					/4
Manus					/4
Carpus					/4
Elbow					/4
Shoulder					/4
Pes					/4
Tarsus					/4
Stifle					/4
Hip					/4

8 Palpation and Manipulation – **Cat Response** (check ALL that apply)

Joint	Flinches/ withdraws	Vocalizes /hisses/ growls	Threatens to bite or scratch	Response is repeatable	Non-evaluable response (ex: aggression)
Cervical spine					
Thoracolumbar spine					
Sacrococcygeal spine					
Manus					
Carpus					
Elbow					
Shoulder					
Pes					
Tarsus					
Stifle					
Hip					

Figure S1. MI-CAT(V)-v1.

MI-CAT(Veterinary)

#	Assessment Criteria		Grade
Assign a value for each of categories 1-4 prior to hands-on examination. The cat should be allowed to walk on exam room floor, be placed on a low bench or chair to observe willingness/ability to jump down, and encouraged to jump up by placing the empty carrier on a bench/chair in front of the cat.			
1	*Exploratory behavior*		
	Walks and/or runs; jumps freely		0
	Walks and runs; jumps with mild encouragement		1
	Walks but does not run; jumps with mild encouragement		2
	Walks and runs; reluctant or unwilling to jump		3
	Walks normally; does not run; reluctant or unwilling to jump		4
	Walks slowly/cautiously, or with abnormal or lowered body posture		5
	Recumbent/sitting (no ambulation)		6
2	*Gait*		
	Normal gait		0
	Not assessable – limited or no ambulation		1
	Occasionally awkward (e.g., misses a jump or missteps)		2
	Mild abnormality (e.g. inconsistent/mild stiffness/weakness or abnormal limb placement/carriage)		3
	Moderate abnormality (e.g. consistent/moderate stiffness/weakness or abnormal limb placement/carriage)		4
	Obviously limping on 1 or more limbs		5
3	*Body Posture*		
	a. head, torso, tail	Ambulates/stands/sits/lies with even weight distribution from front to rear, back level, head up, tail above horizontal	0
		Head low/tail lowered (not tucked)	1
		Overt abnormalities: weight shifted forward or backward, hunched back, limp tail<br>1 finding	2
		≥2 findings	3
	b. front limbs	Ambulates/stands/sits/lies with limbs in normal state of flexion/extension, even weight distribution from right to left	0
		Overt abnormalities: limb hyperflexion, limb hyperextension, unequal weight distribution from right to left, or other asymmetry<br>1 finding	1
		≥2 findings	2
	c. rear limbs	Ambulates/stands/sits/lies with limbs in normal state of flexion/extension, even weight distribution from right to left	0
		Overt abnormalities: plantigrade stance, limb hyperflexion or hyperextension, unequal weight distribution from right to left, or other asymmetry<br>1 finding	1
		≥2 findings	2

Figure S2. *Cont.*

4	*Interactions with examiner*		
	Friendly: approaches/rubs/wants to be petted		0
	Neither avoids nor solicits contact		1
	Withdraws/avoids touch		2
	Hisses/growls/swats/bites or threatens		3
Perform palpation and manipulation in whatever position is best tolerated by the patient. Assess whether response is present and repeatable, and association with pain. A response may consist of: tensing, flinching, withdrawing or attempting to escape, vocalization (hiss, growl, meow, etc.), turning toward the handled body part or threatening to bite or scratch, or biting or scratching.			
**5**	*Cat response to palpation and manipulation*		
	a. cervical spine	No repeatable response	0
		Repeatable response, but not clear if due to pain	1
		Repeatable painful response	2
	b. thoracic spine	No repeatable response	0
		Repeatable response, but not clear if due to pain	1
		Repeatable painful response	2
	c. lumbar (and lumbosacral) spine	No repeatable response	0
		Repeatable response, but not clear if due to pain	1
		Repeatable painful response	2
	**Total score: axial skeleton**		/6
	d. carpus	No repeatable response	0
		Repeatable response, but not clear if due to pain	1
		Repeatable painful response	2
	e. elbow	No repeatable response	0
		Repeatable response, but not clear if due to pain	1
		Repeatable painful response	2
	f. shoulder	No repeatable response	0
		Repeatable response, but not clear if due to pain	1
		Repeatable painful response	2
	**Total score: front limb**		/6
	g. tarsus	No repeatable response	0
		Repeatable response, but not clear if due to pain	1
		Repeatable painful response	2
	h. stifle	No repeatable response	0
		Repeatable response, but not clear if due to pain	1
		Repeatable painful response	2
	i. hip	No repeatable response	0
		Repeatable response, but not clear if due to pain	1
		Repeatable painful response	2
	**Total score: rear limb**		/6
Add scores for 1, 2, 3a-c, 4, 5 Total axial skeleton, Total front limb, Total rear limb			
**Total MI-CAT(V) score**			**/ 39**

Figure S2. MI-CAT(V)-v2.

© 2015 by the authors; licensee MDPI, Basel, Switzerland. This article is an open access article distributed under the terms and conditions of the Creative Commons Attribution license (http://creativecommons.org/licenses/by/4.0/).
